# National study of colorectal cancer genetics

**DOI:** 10.1038/sj.bjc.6603997

**Published:** 2007-09-25

**Authors:** S Penegar, W Wood, S Lubbe, I Chandler, P Broderick, E Papaemmanuil, G Sellick, R Gray, J Peto, R Houlston

**Affiliations:** 1 Section of Cancer Genetics, Institute of Cancer Research, Sutton, Surrey SM2 5NG, UK; 2 Department of Cellular Pathology, St George's Hospital, London, SW17 0QT, UK; 3 Birmingham Clinical Trials Unit, University of Birmingham, Birmingham, UK; 4 Non-Communicable Disease Epidemiology Unit, London School of Hygiene and Tropical Medicine, London, UK; 5 Institute of Cancer Research, Surrey, UK

**Keywords:** genetics, colorectal cancer, predisposition

## Abstract

Approximately, a third of all colorectal cancer (CRC) is due to inherited susceptibility. However, high-risk mutations in *APC*, the mismatch repair (MMR) genes, *MUTYH/MYH*, *SMAD4*, *ALK3* and *STK11/LKB1* are rare and account for <5% of cases. Much of the remaining variation in genetic risk is likely to be explained by combinations of more common gene variants that modestly increase risk. Reliable identification of such ‘low penetrance’ alleles would provide insight into the aetiology of CRC and might highlight potential therapeutic and preventative interventions. In 2003, the National Study of Colorectal Cancer Genetics (NSCCG) was established with the aim of collecting DNA and clinicopathological data from 20 000 CRC cases and a series of spouse/partner controls, thereby creating a unique resource for identifying low-penetrance CRC susceptibility alleles. The National Cancer Research Network (NCRN) adopted NSCCG onto its portfolio of trials and 148 centres in the United Kingdom (UK) are now actively participating. Over 8700 cases and 2185 controls have so far been entered into NSCCG. Our experience in developing NSCCG serves to illustrate how world-class DNA databases for genetic analyses can be rapidly developed in the United Kingdom.

Colorectal carcinoma (CRC) is a major cause of cancer-related mortality in Western countries (Parkin *et al*, 2003). Evidence from twin studies indicates that inherited susceptibility is responsible for ∼30% of CRC (Lichtenstein *et al*, 2000). While germline mutations in *APC*, the mismatch repair (MMR) genes, *MUTYH/MYH*, *SMAD4*, *ALK3* and *STK11/LKB1* confer a high risk of CRC, collectively, such mutations account for <5% of disease (Aaltonen *et al*, 2007) with much of the remaining variation in genetic risk likely to be explained by combinations of more common, lower penetrance variants. This ‘common disease–common variant’ hypothesis implies that conducting association analyses based on scans of single nucleotide polymorphisms (SNPs) should be a powerful strategy for identifying low-penetrance genes (Risch and Merikangas, 1996; Botstein and Risch, 2003). Such an assertion is supported by recent data showing an association between 8q24 variants and CRC risk (Haiman *et al*, 2007; Tomlinson *et al*, 2007; Zanke *et al*, 2007).

Despite much research, few definitive low-penetrance susceptibility alleles have been unequivocally identified through association studies. As with many other diseases, positive associations have been reported for various polymorphisms of genes such as *NAT2, GSTM1*, *CYP1A1*, *STK15* and *TP53* from small studies, but few of the initial positive results have been replicated in subsequent studies (Vinesis *et al*, 1999; Houlston and Tomlinson, 2001; Ye and Parry, 2003; Borlak and Reamon-Buettner, 2006; Webb *et al*, 2006a). The inherent statistical uncertainty of case–control studies involving just a few hundred cases and controls seriously limits the power of such studies to identify reliably genetic determinants conferring modest but potentially important risks. Hence, the identification of genes associated with CRC predisposition and determination of their contribution to disease incidence is, however, contingent on having DNA samples from large, systematic series of cancer patients.

The National Cancer Research Network (NCRN) was established to provide support for clinical cancer research in England and is one of the most substantial and constructive developments in the area of cancer research to be made in the United Kingdom in recent years. The NCRN is made up of 40 geographically distinct Networks covering the entire country (33 in England, 3 in Scotland, 3 in Wales and 1 in Northern Ireland). Within each Network there are clinical research support staff and infrastructure to promote accrual of patients into trials and studies, and the collection of high-quality clinicopathological data and appropriate biological samples. Hence, the NCRN presents a major scientific initiative not only in the field of clinical trials but also in the field of genetic epidemiology.

To create a resource for identifying low-penetrance predisposition alleles for CRC, we established the National Study of Colorectal Cancer Genetics (NSCCG) in March 2003 to collect DNA and clinicopathological data from 20 000 cases. Through linkage with the NCRN, it has been possible to create a world-class resource with samples from over 8500 patients in less than 4 years.

## MATERIALS AND METHODS

### Eligibility criteria

All patients with colon or rectal cancer (International Classification of Diseases (ICD) ninth edition codes 153 and 154, respectively) within 5 years of diagnosis were deemed eligible for the study. To ensure that data and samples were only collected from bona fide adenocarcinoma cases histological confirmation of disease was a prerequisite. The upper limit was initially 75 years at diagnosis, but this was subsequently reduced to 69 years in the light of excellent ascertainment to increase the proportion of younger patients whose cancers are more likely to be due to inherited susceptibility (Houlston and Peto, 2004). Coupled with patient recruitment, spouses/partners who had no known past or current history of malignancy were invited to participate for the purposes of contributing to the generation of a control series. No age limit was imposed for these individuals.

### Procedural outline

A standardised questionnaire administered to patients is being used to collect demographic characteristics – sex, date of birth, ethnic group (White, Black-Caribbean, Black-African, Black-other, Indian, Pakistani, Chinese, and Other), country of birth and current area of residence. In addition, detailed identifiable information is being collected on all first-degree relatives (parents, siblings and offspring) on cancer diagnoses. An open question is being used to illicit information on cancer diagnoses in second-degree relatives. All questionnaires are self-administered and no surrogate responders were used. Clinicopathological details of CRCs (site, stage and grade at presentation) were obtained from the referring clinician using a standardised registration form. For controls, details of sex, date of birth, ethnic group, place of birth and current area of residence were collected through a self-administrated questionnaire. Ethylenediaminetetraacetic acid–venous blood samples (10–20 ml) were collected from all participants. In addition to blood samples, archival blocks representative of patients' CRC were routinely being sought.

Consent forms, questionnaires, registration forms and blood samples were returned to the Institute of Cancer Research (ICR) by mail. Blood samples collected were stored at −80°C prior to DNA extraction and quantification. Extraction of genomic DNA from whole blood is being undertaken by means of either a conventional salt lysis procedure or Chemagic Magnetic Separation Module 1™ methodology.

Colorectal cancer develops along at least two biologically distinct pathways characterised by genetic abnormalities, which can reflect different underlying predispositions (Ilyas and Tomlinson, 1996). The chromosome instability pathway is typified by chromosomal abnormalities resulting in loss of heterozygosity and tumour suppressor gene inactivation, while cancers exhibiting microsatellite instability (MSI) have defective DNA mismatch repair. In view of this, we are routinely evaluating CRCs for MSI using the following methodology: 10 *μ*m sections are cut from formalin-fixed paraffin-embedded tumours, lightly stained with toluidine blue, and regions containing at least 60% tumour are micro-dissected. Tumour DNA is being extracted using the QIAamp DNA Mini kit (Qiagen, Crawley, UK) according to the manufacturer's instructions and genotyped for the mononucleotide microsatellite loci BAT25 and BAT26, which are highly sensitive markers of MSI (Zhou *et al*, 1998). Samples showing novel alleles at either BAT26 or BAT25 or both markers are assigned as MSI (corresponding to a high level of instability, MSI-H Boland *et al*, 1998).

### Statistical considerations

The primary aim of establishing NSCCG was to generate a DNA resource of CRC patients of sufficient size to robustly identify low-penetrance alleles by association studies of genetic polymorphisms. From the outset, it was envisaged that such searches would be conducted on a genome-wide basis. It is well-recognised that as such studies involve genotyping a vast number of markers, a large number of false-positive associations will inevitably be generated and only a small number will be truly associated with disease susceptibility. Hence, associations need to attain a high level of statistical significance to be established beyond reasonable doubt and significance levels of ∼10^−7^ have been proposed as being appropriate (Risch and Merikangas, 1996). The original target of NSCCG was to assemble a series that will include 2000 cases. This figure had been based upon contemporaneous preconceptions on the probable impact of common alleles on disease risk. Recent data from association studies, however, indicate that common disease alleles are likely to be associated with risks typically <1.5 (Easton *et al*, 2007; Gudmundsson *et al*, 2007; Haiman *et al*, 2007; Tomlinson *et al*, 2007; Zanke *et al*, 2007). To identify alleles conferring such risks to have 80% power-stipulating statistical thresholds of 10^−7^ necessitates having access to sample sets of at least 10 000 cases and 10 000 controls. These statistical considerations led us to significantly revise our target sample size to ensure that we would generate a resource adequately powered to identify such disease-causing alleles and to examine for epistatic interactions.

### Ethical considerations

In generating DNA registries, ethical considerations are central to study design. One of the particular strengths of studies such as NSCCG is that once constructed the DNA database can be probed repeatedly for different existing and newly identified candidate risk factor genes. It is not feasible to contact all study entrants to seek further written consent for each specific test, therefore, the information sheet study discussion and consent is centred on the general concept of ‘genetic analyses’. As these investigations were to be solely for research to find new gene(s) predisposing to cancer, it was implicit that no individual results will be conveyed to persons. In publications of findings no study entrant would be identifiable. As with all studies of this nature, we clearly stated that if a study entrant wished to withdraw, then their DNA sample and all information held on them would be destroyed. To ensure confidentiality, data are held under secure conditions at the Institute of Cancer Research (ICR) in accordance with the Data Protection Act (1998).

All clinical information and biological samples were obtained only after fully informed consent was obtained from participating individuals, and in accordance with the tenets of the Declaration of Helsinki. Ethical approval for the study was obtained from the Multi-Centre Research Ethics Committees (MREC/98/2/67; MREC02/0/97).

## RESULTS

**Figure 1:**
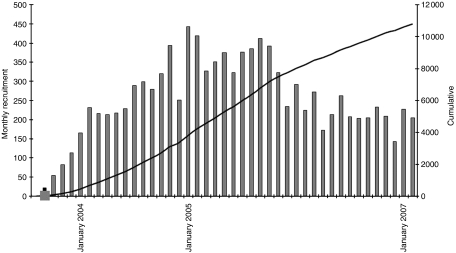
Accrual rate of patients and controls to NSCCG.

**Figure 2:**
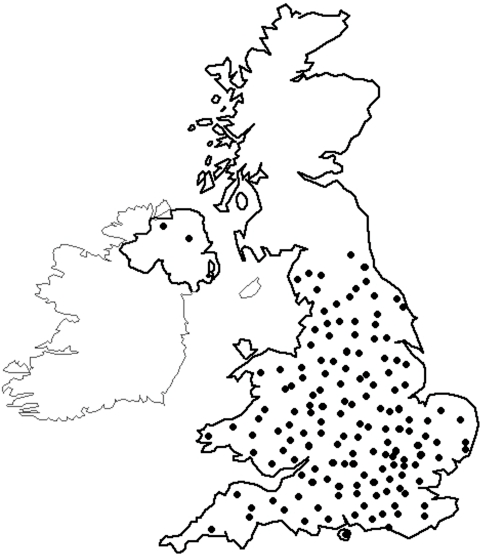
Sites of centres in the United Kingdom recruiting to NSCCG as of March 2007.

After securing ethical permissions at national level through the Multi-Centre Research Ethics Committee, the study was incorporated into the NCRN (National Cancer Research Network) portfolio in March 2003. It was subsequently rolled out across England after individual hospitals had obtained relevant local ethical permissions. As the number of centres becoming active participants in NSCCG widened, accrual of both patients and controls significantly increased (Figure 1). Currently, 148 sites are participating in NSCCG (Figure 2) and recruitment stands at approximately 160 cases and 40 controls per month. Although most hospitals entering subjects into NSCCG are located in England, centres in Wales and Northern Ireland are now participating and a number in Scotland are in the process of gaining local ethical permissions to participate, thereby, making the study UK-wide.

To date, blood samples and completed questionnaires have been collected from 8722 cases and 2185 controls. The NSCCG collection will continue until samples from 20 000 CRC cases has been assembled. The number of male patients ascertained or included (59% of cohort) reflects the slight sex preponderance of the disease. In terms of ethnicity, over 90% of patients entered into NSCCG were self reported as White Caucasians. Although controls were of similar age in comparison to cases (mean age 59 years; s.d.=9.5) and 90% were self-reported as White Caucasians not surprisingly, the majority (64%) were female.

A high proportion of the cases have been diagnosed at a young age, reflecting the criterion for ascertainment (Table 1) ; specifically, 29% of the cases were aged <55 years old at diagnosis, compared with <10% in the general population. Approximately 15% of patients have a family history of CRC defined by having at least one affected first-degree relative. The 60 : 40 ratio of colonic (ICD 153) to rectal disease (ICD 154) of cases recruited to date is in keeping with that observed in the general UK population (Table 1). Similarly, stage at presentation is comparable to that generally observed. To date, 3088 tumour blocks have been collected and 2516 evaluated for MSI; 12% showing MMR deficiency, concordant with the frequency of this phenotype in unselected CRC (Storojeva *et al*, 2005).

## DISCUSSION

Over the coming years our expanding knowledge of cancer genetics will have a major impact on our ability to predict an individual's level of risk of developing cancer, detect and diagnose cancer early and select treatments which are most likely to be effective. Ultimately the genetic revolution may lead to ways of preventing cancer. Advances in genetics will lead to a greater understanding of inherited susceptibility to cancer. The relative influence of genes on cancer development is variable and ranges from situations where genetic factors predominate and are highly predictive of disease development, to others where they play only a minor role in modifying the effect of environmental exposure to toxic substances.

(NHS Cancer Plan, 2000)

Following the sequencing of the human genome, large-scale harvests of SNPs have been conducted, and there are currently >10 million documented human SNPs (dbSNP). Patterns of linkage disequilibrium (LD) between polymorphisms have been characterised allowing subsets of tagging SNPs to be selected that, through LD with other variants, capture a large proportion of the common sequence variation in the human genome. These comprehensive sets of tagging SNPs, which capture most of the common sequence variation, coupled with the development of highly efficient analytical platforms allow whole-genome studies for disease associations to be conducted cost effectively. This approach is unbiased and does not depend upon prior knowledge of function or presumptive involvement of any gene in disease causation. Moreover, it avoids the possibility of missing the identification of important variants in hitherto unstudied genes or regions of the gene-harbouring and gene-controlling elements. The relationship between patients' genotype and aetiology of cancer is, thus, now open for further exploration.

Here, we have demonstrated that the centralisation of cancer services in the United Kingdom offers an opportunity to establish large, well-characterised cohorts by targeting collection to the largest centres. Moreover, mobilising NCRN networks provides a means of consistently delivering the data and sample collection needed to complete genetic epidemiology studies on the required scale.

Ascertainment of cases through NCRN has been directed towards those diagnosed at young age and this serves to empower the resource for identifying disease-causing alleles by virtue of genetic enrichment. The possibility of population stratification leading to false inference of disease–genotype association can be addressed by adjusting for known region/ethnicity or by using information on unlinked genetic markers. In NSCCG, the geographical area of birth and area of residence within the United Kingdom is known for all of the individuals, and this information can be used to allow analyses stratified by region of residence, reducing any effects of population stratification.

For some analyses, family history information on cancer diagnosis in relatives is required. In our study, participants are providing information on cancer diagnosis in relatives without verification of the diagnosis through medical sources or health records. Subject to ethical approval, there is a possibility of verifying cancer diagnoses verified through national registries. While such verification is theoretically desirable since studies have shown that CRC patients correctly report ∼80% of positive family histories and >95% of negative family histories in first-degree relatives (Aitken *et al*, 1995; Ziogas and Anton-Culver, 2003), self-reported data still has great utility and is unlikely to systematically bias many types of analyses.

As blood samples have been ascertained from many clinical centres in the United Kingdom, we are cognisant of the potential problem of differential bias in genotyping. We have however, no evidence of differences in DNA quality as we have previously documented call rates of 99.8% in 1526 of samples ascertained through NSCCG (Webb *et al*, 2006b).

The NCRN research networks are established within cancer care networks where access to partners is readily available and direct. They are not designed to collect samples from the general population or the unrelated population so our choice of collecting samples from partners was a pragmatic one appropriate for the NCRN. Inevitably, in studies such as NSCCG, a smaller number of samples from controls will be collected than from cases since in addition to lack of compliance many patients do not have a current partner. While the sex of controls ascertained through initiatives such as NSCCG will usually be of the opposite gender to cases by pooling samples from other similar studies, it is possible to develop large control data sets such as a 1958 Birth Cohort established in the United Kingdom currently being used by the Wellcome Trust Case Control Consortium (WTCCC). Because of the difficulty of obtaining sufficiently detailed and accurate data on environmental exposure in studies such as NSCCG, and because there are issues to do with comparability of exposure data from controls assembled from different studies, it is acknowledged that studies of environmental risk factors including gene–environment interaction will be limited in resources such as NSCCG.

Given the projected size of NSCCG, the main value of collections such as this will be in studies of common genetic risk factors and gene–gene interactions, hypotheses regarding gene–environment interaction will require alternative data sets. Accepting the limitations of NSCCG, our experience in developing this resource serves to illustrate how large DNA databases for genetic analyses can rapidly be developed in the United Kingdom.

## Figures and Tables

**Table 1:** Clinicopathological characteristics of colorectal cancer patients recruited to the National Study of Colorectal Cancer Genetics

	**Number of patients (%)**
Total	8722
Gender (male/female)	5175 (59%):3547 (41%)
	
*Age at diagnosis (years)*	
<30	63 (0.1%)
31–35	75 (0.8%)
36–40	174 (2.0%)
41–45	329 (3.8%)
46–50	613 (7.0%)
51–55	1234 (14.2%)
56–60	1981 (22.7%)
61–65	2316 (26.6%)
66–69	1745 (20.0%)
70+	192 (2.2%)
Mean (SD), Median	58.8 (8.4), 60
	
*Ethnicity*	
Asian	59 (0.7%)
Bangladeshi	1 (<0.1%)
Black-African	20 (0.2%)
Black-Caribbean	56 (0.7%)
Black-Other	8 (<0.1%)
Indian	69 (0.8%)
Jewish-Ashkenazi	18 (0.2%)
Jewish-Sephardic	1 (<0.1%)
Pakistani	13 (0.2%)
White	8477 (97.2%)
	
*Type of colorectal cancer*	
Colonic disease (ICD-9 153)	5247 (60%)
Ascending	747
Hepatic flexure	159
Splenic flexure	178
Transverse colon	391
Descending colon	338
Sigmoid colon	2337
Caecal	1035
Other/not specified	62
Rectal disease (ICD-9 154)	3475 (40%)
Recto-sigmoid junction	509
Ampulla	2962
Other/not specified	4
